# Book Review

**Published:** 1903-03

**Authors:** 


**The Evolution of Artificial Mineral Waters.** By WILLIAM KIRKBY, F.L.S. Pp. x., 155. Manchester: Jewsbury & Brown. 1902.—This monograph describes the initiation and development of the mineral-water industry. There are no technical descriptions of machines and processes, and no formulæ for preparations, so that it is not intended as a manufacturer's handbook, but it is intended to convey to the ordinary reader an intelligent and interesting account as to how artificial mineral waters came into being, and how they are manufactured to-day. Appended is a series of photographic views of the manufactory of Messrs. Jewsbury and Brown at Ardwick Green, Manchester.

**Menstruation and its Disorders.** By Arthur E. Giles, M.D. Pp. 100. London: Baillière, Tindall & Cox. 1901.—In this handbook the author gives a very full account of the physiology and pathology of menstruation. In dealing with the physiological phenomena, he quotes largely from the investigations of Walter Heape on the menstrual phenomena in the female of semnopithecus entellus. He points out that menstruation in the human female corresponds to the pro-œstium in the other mammalia, except in apes, in whom the function corresponds with that in the human female. He gives a very concise account of the different conditions associated with amenorrhœa, under which heading he does not include cases of retained menses, which he calls crypto-amenorrhœa, which strikes us as a logical arrangement. He, in our opinion, rightly discards the idea of vicarious menstruation, describing the hemorrhage usually called by this name as "supplementary hemorrhages." We notice one or two rather important omissions: one the failure to mention the condition often termed "adenoma benignum" as a cause of both menorrhagia and metrorrhagia; in the other the failure to emphasise the frequency of ovarian adhesions and cystic degeneration without great enlargement as causes of dysmenorrhœa. This latter point deserves fuller notice. He objects to the use of the term "obstructive" as usually applied to dysmenorrhœa, and in this we consider he is logical: "spasmodic" is both more correct, and does not indicate pathological ideas. He also gives some interesting statistics showing the relation between the age of menstruation and sterility. Though on the whole the statements made are sound, we should have preferred that the author had left less to the imagination of the reader, and avoided an indefiniteness at times rather annoying.

**Points of Practical Interest in Gynæcology.** By H. Macnaughton-Jones, M.D. Second Edition. Pp. ix, 157. London: Baillière, Tindall & Cox. 1901.—In a review of the previous edition of this little work we commented on the practical hints given by the author, and do not find it necessary to again refer to the many good points he impresses on the reader. The work is especially valuable in that it is almost a collection of clinical lectures, and is what it is stated to be—a collection of practical points in maxims of, and hints on, gynæcology. The author has added a chapter on retroversion of the uterus, and shows how he has become gradually converted to the treatment of this affection by operation. He acknowledges that in many cases he has been able to cure patients by minor measures, *i.e.* by local absorbent, massage, posture, curettage, and pessaries, even though at first sight the condition seemed incurable, and then proceeds: "But recalling the numbers in whom there was no such satisfactory issue, the time, the suffering and inconvenience involved in the process, I should not now submit them to the same treatment, but advise operation." He considers that in cases where the uterus is mobile and adnexal disease absent, Alexander's or the Alexander-Adam's operation is the most suitable. If adnexal disease be present cœliotomy is the best course, in his opinion. His last paragraph will meet with universal acceptance: "Displacements that do not yield to such palliative treatment that pessaries fail to cure, that are complicated by disease of the adnexa, that cause symptoms seriously interfering with the health of the woman, and which prevent her following her avocation, demand operative interference."

**Advice to Women on the Care of the Health before, during, and after Confinement.** By FLORENCE STACPOOLE. Third Edition. Pp. viii., 184. London: Cassell and Company Limited. 1901.—This little work we can recommend: it is full of common-sense maxims and useful hints as to the conduct of women during pregnancy in order that they may maintain their health. The hints as to the preparation necessary for parturition are most sound and valuable. We feel, however, that we cannot commend the insertion of prescriptions to be used by the public. This, in our experience, is a fertile source of work for the doctor, and therefore not conducive to the true interests of the patient. If the author can disabuse the minds of the female public of some of the numerous harmful superstitions prevalent with regard to pregnancy and labour, she will be doing an immense service.

**Transactions of the Obstetrical Society of London.** Vol. XLII. London: Longmans, Green, & Co. 1901.—This volume, as usual, contains an abundance of interesting communications from which the following may be selected as being especially worthy of notice, either on account of their originality or practical value. Dr. Spencer contributes an account of "Four cases of Rupture of the Uterus successfully treated by packing the tear *per vaginam* with iodoform gauze." With the experience now common to many that the opening of the peritoneal cavity *per vaginam*, even in circumstances unfavourable to strict asepsis, *e.g.*, in cases of sloughing carcinoma, is usually unattended by serious results, the success of Dr. Spencer's plan would not be surprising if viewed on merely general grounds, and in cases in which the tear had not opened the peritoneal cavity, might be generally expected. When, however, we find that in two cases the peritoneal cavity was not only opened freely, but contained much blood, and in one "a piece of slightly offensive membrane" was hanging in the vagina, the successful use of this method of treatment is sufficiently startling. It is of course only another illustration of the self-protective power of the peritoneum when free drainage is used. Dr. Spencer's results in these four cases contrast most favourably with the results obtained by him in other cases treated by operation—in every case unsuccessfully. Several other papers and specimens referring to this subject will be found in the volume. The "Causation of Sex" has for long troubled the minds of speculative enquirers. Dr. Rumley Dawson is of opinion that ova from the right ovary produce males, and those from the left ovary females, and mentions a number of curious coincidences in the way of occluded tubes, corpora lutea, and the sex of the children connected with these phenomena. He dismisses the cases mentioned by those of his audience who discussed the paper, in which the removal of one ovary had been followed by the production of children of both sexes, as due to the incomplete removal of the ovary. Although this argument may apply in cases of oöphorectomy with adhesions present, we fail to see how it can in a case which is within our knowledge, in which ovariotomy for a large cystic tumour before marriage has not prevented the birth of a family of two girls and two boys. Other interesting papers may be mentioned: "A case of Double Tuberculous Pyosalpinx with intercommunication of the tubes," by Dr. Galabin; also a similar condition described by Mr. Malcolm; a case of "Repeated Ectopic Gestation," by Dr. Lewers. Space will not allow of detailed reference to the many other interesting papers.

**Pediatrics.** THOMAS MORGAN ROTCH, M.D. Third Edition. Pp. 1021. Philadelphia and London: J. B. Lippincott Company. 1901.—This is a well-printed and well-illustrated book, but one cannot help feeling some sense of disappointment after its perusal. It contains few facts about disease in children that cannot be gathered from a good text-book of general medicine, and one would have thought the great experience of Dr. Rotch would have enabled him to make more practical suggestions as to treatment. The feeding of infants, as one would expect, is dealt with very fully. As is well known, Dr. Rotch and some of his confrères have aimed at gauging the nutritive needs of any particular infant with mathematical accuracy, and of prescribing amounts of fat, proteid, and sugar accordingly. The attempt is praiseworthy, and, although to most of us the remark of Jacobi may forcibly appeal, that "the best thing to mix with milk is a little common-sense," we cannot but think that, medically at least, common-sense must be based upon scientific accuracy. Not uncommonly, however, as perhaps in this case, scientific zeal may override sound judgment, yet even should that be so, we may repeat that the attempt to feed infants on a definite system suited to every individual case is praiseworthy.

**Forensic Medicine and Toxicology.** By J. DIXON MANN, M.D. Third Edition. Pp. xii., 692. Charles Griffin & Company, Limited. 1902.—Very little need be said in praise of this standard work, which has now reached a third issue. Besides a general revision, notices of some additional poisons have been added, and the book now stands in the very highest rank of contemporary treatises on the subject. Bulky as the volume is, the text has been compressed more than is desirable, and we only wish that more cases had been given illustrating obscure points. The account of the late epidemic of arsenical poisoning is much too brief, though it is possible that the full meaning of the outbreak is yet undecided. Chronic alcoholic poisoning should have a separate section, if only for the sake of diagnosis. The author does not refer to the treatment of carbolic acid poisoning by washing out with alcohol, though apparently successful cases have been reported. Of late, too, a great deal of work has been done on the investigation of lead poisoning, especially that which is caused in the manufacture of earthenware. The danger to the operatives, unless various precautions are taken, is well known, and the mortality has already been greatly reduced. Whether it will be possible to use leadless glaze universally is less certain, and it becomes an important question whether the common use of crockery covered with a leaden glaze may not be the cause in susceptible persons of much of the existing kidney and nerve disease, or even of some of those affections which have been ascribed to "uric acid." There would be great difficulties in the detection of the poison in such chronic cases, though in the early stages of plumbism certain changes in the red cells have been recently demonstrated. The Widal test should be made in examining supposed cases of starvation to exclude the possibility of the symptoms being due to recent ambulatory typhoid; and in the examination of bloodstains, the new method of distinguishing human from animal blood based on the Widal reaction should be noticed in the next edition. On the whole, the student and practitioner will probably find no better book than this as a guide to a difficult subject.

**Medical Guide to the Hot Mineral Baths of Bath.** Pp. 111. Bath: The *Herald* Office. 1902.—This guide, with extracts from the Report of the Special Commission of the *Lancet*, has been published by the Baths Committee of the Bath Corporation, with the approval of the Local Council of the British Medical Association. It has therefore received an official recognition as an authoritative account of what can be done by the Bath waters, and the personal element has been entirely eliminated. The aim of the work is to show "that far from living upon its historic past, Bath stands to-day unsurpassed by any other health resort, at home or abroad, whether in regard to the medicinal virtues of its mineral springs, or the luxury and scientific completeness of the several establishments wherein the healing waters are administered." An appendix, containing a list of hotels, boarding and lodging houses is likely to be useful.

**Heath's Practical Anatomy.** Ninth Edition. Edited by J. Ernest Lane. Pp. xviii., 696. London: J. & A. Churchill. 1902.—This well-known manual has now reached its ninth edition. The aim of the Editor has been to bring the book up to date, and to add new drawings and diagrams without increasing the dimensions of the volume, but at the same time in no way to impair its utility. This he has succeeded in doing. The references to surgery, which are unnecessary in a work of this sort, and some of the old illustrations, have been omitted. We note several new illustrations which will be of much value to students; we might mention in particular four very clear diagrams, showing the arrangement and principal anastomoses of the arteries of the neck and extremities, which are a considerable acquisition to the work. Another very useful addition is a tabular enumeration of the arteries and nerves, which is appended to the dissection of each region of the body —thus showing at a glance the distribution of all the branches, and their connection with the main trunks. We do not understand the sub-division of the portion of the prostate anterior to the urethra, as figured in a sagittal section of that gland (fig. 167). A transverse fissure is there represented as dividing this portion into two equal parts. The series of coloured plates, which were hitherto placed together at the beginning of the book, have now been interpolated through the volume in the description of the regions to which they refer. The general plan of the work and the excellent illustrations are too well known to need description here. As a dissecting-room manual it is portable and convenient, containing in one volume all the information that is necessary. The author does not profess to provide an elaborate treatise, but a concise and accurate account of all that the student will require to know for ordinary examinations, and as such it will commend itself to many students.

**The Principles and Practice of Medicine.** By William Osler, M.D., F.R.S. Fourth Edition. Pp. xvii., 1182. Edinburgh: Young J. Pentland. 1901.—Such a well-known and widely-read text-book needs no further recommendation, but we may point out that the last edition is not in any sense a mere reprint of its predecessor. Thus the article on typhoid fever has been in great part re-written, the subject of malaria recast, and many new articles have been added to bring this excellent text-book thoroughly up to date. Further comment is unnecessary, for the name of the author guarantees that the new matter is as reliable and well written as the old.

**The Diagnosis and Modern Treatment of Pulmonary Consumption, with especial reference to the Early Recognition and the Permanent Arrest of the Disease.** By Arthur Latham, M.D. Oxon. Pp. 215. London: Baillière, Tindall and Cox. 1903.—The author of the prize essay on the erection of his Majesty's sanatorium may well claim to be an authority on the modern views with regard to tubercular disease. This volume is a series of articles previously published, but now collected in a form which should prove of service as a practical guide to the diagnosis and treatment of pulmonary consumption. Those who wish to study the principles of open-air treatment as carried on either in a sanatorium or in a private house will derive every assistance from a study of this standard account of the subject. The author does not, however, ignore other aids to treatment, medicinal and dietetic.

**The Treatment of Injuries by Friction and Movement.** By WHARTON P. HOOD, M.D. Pp. vi., 182. London: Macmillan & Co., Limited. 1902.—Dr. Wharton Hood has made such a reputation for himself in the treatment of certain injuries of the limbs and joints by so-called "bone-setting" that no explanation is needed from him as to his reasons for writing this book. He tells us, however, that he was indebted to the noted Mr. Hutton, for disclosing to him the "secrets of his art." These secrets had been handed down for a very long period. Mr. Hutton came of a family which had practised a rude surgery among the dalesmen of the North of England for many years, and had acquired considerable skill in curing a certain class of injuries without requiring the patient to rest. He always told the patient "a bone was out," and then adopting various devices of grasping and of position, by which the muscles were hindered from assuming an attitude of resistance, he suddenly bent, extended, or rotated the limb, keeping at the same time firm thumb pressure upon the tender spot; usually something would be heard to snap or crack, and freedom of movement and freedom from acute pain would at once result. Dr. Wharton Hood not only carries out the method of Hutton in treating chronic injuries, but in this book he strongly urges the treatment of recent fractures without splints, by means of strapping plaster and constant movements. We will not describe his methods here, but we recommend all surgeons to read the book. There is much in it with which we agree, and much with which we disagree. Thus, when he insists that local swelling and blood-stasis, the results of a recent fracture, may be entirely removed by gentle movements of stroking or squeezing, directed from the distal towards the proximal portion of the limb, together with regular movements of the joints carried out by the patient's own efforts, without any fixation, we quite agree with him; but when he tells us that in treating fractures of the shaft of the femur the patient should be allowed to flex the knee on the fifth or sixth day, and that in fractures of the patella the patient should only be confined to bed for a day or two, and that no attempt should be made to bring the fragments into apposition, we do not agree with him.

**The Study of the Pulse.** By JAMES MACKENZIE, M.D. Pp. xx., 325. Edinburgh: Young J. Pentland. 1902.—The standpoint of the author is revealed in the following sentences from the preface: "Whilst I may have occasion to refer to what I consider the erroneous view of others, I am conscious that where so many more competent observers have erred, it is but likely that I should go astray. In writing this work the necessity therefore has been kept distinctly in view of giving a clear conception of the facts as distinct from the theories." The illustrations are very numerous, and throughout the work the author constantly refers to sphygmographic tracings to support the views enunciated. Many questions of practical interest are ably discussed. Such, for instance, as the supposed delayed radial pulse resulting from aneurysm of the aorta, etc., which the author demonstrates very clearly to be a fallacy. The work is worthy of a high place in medical literature.

**Guy's Hospital Reports.** Vol. LVII. London: J. & A. Churchill. 1902.—Dr. W. Hale White describes two cases of acute rheumatoid arthritis, and, like many other observers, concludes that it is a separate disease, which has no association with the chronic forms. Dr. Frederick Taylor and Mr. Arbuthnot Lane record two cases of volvulus, treated by resection of the twisted bowel. One of these cases was successful, but it is to be hoped that this will not induce others to follow a practice which must be more dangerous than the simpler method of first forming an artificial anus and draining the distended bowel. Various other papers make up a volume of more than usual excellence.

**Transactions of the Medical Society of London.** Vol. XXV. London: Harrison and Sons. 1902.—This record of the 129th session of the Medical Society of London contains the usual assortments of papers on many subjects, and is a record of much original work, and of views which have not yet found their way into the text-books. The debate on cardiac pain, which was opened by Dr. Markham Skerritt, was of great interest, and is worthy of renewed study. It directs especial attention to the fact that "pain that is the expression of the distress of the heart under too heavy a load of work is met with in all degrees of intensity, from slight and trivial discomfort at the one extreme up to the overwhelming agony of the classical angina pectoris at the other. There is no line of demarcation, either clinical or pathological; the distinction is one of degree only, and not of character." The annual oration, by Dr. Stephen Mackenzie, reviewed "the powers of natural resistance, or the personal factor in disease of microbic origin," and concluded with the remark that "we must never forget that the *soil* is of as great importance to study as is the *seed* in the proper understanding and treatment of diseases of microbic origin."

**Edinburgh Medical Journal.** New Series. Vol. XII. Edinburgh: Young J. Pentland. 1902.—The most noteworthy contributions of this volume are the series of Morison Lectures, by the Editor, Dr. G. A. Gibson, on "The Nervous Affections of the Heart." After a careful survey of the character and nature of sensory disturbances of the heart, he observes that these sensory affections of the circulation are, in truth and in deed, wonderfully amenable to careful management. A study of the author's experiences in cardiac therapeutics will give most readers an increased power of arresting death, and of guiding patients through critical periods which, if neglected, must end in death, but which, after the crisis is over, may not recur for years.

**Rough Notes on Remedies.** By Wm. Murray, M.D. Fourth Edition. Pp. xi., 176. London: H. K. Lewis. 1901.—This edition of a work which is at least attractive, even if the writer's dicta are not altogether accepted, is enriched with some "annotations to correct misapprehensions, and to make the meaning in the original text clearer." Thus the treatment of chorea by fifteen-minim doses of Fowler's solution has been continued for two or three weeks by some readers with unfortunate results. The author points out that if it does not act as a cure in a week it must be stopped. Three such doses a day may be given during meals to a child of 12 or 15 years when chorea is present, but it must be stopped in a week or sooner if irritation is set up. He finds that arsenic, which he recommended in diabetes, fails in some cases and succeeds admirably in others. The latter seem chiefly those which follow on worry and overwork, while in gouty glycosuria and in diabetes of young people it is rarely of use. He advocates as warmly as ever his large doses of blue pill in some forms of heart disease, and adds that many correspondents attest its value. As to another remedy, nitrate of silver in epilepsy, he has been rather disappointed. Since so many cases do well on bromides, with strontium bromide or borax added, he now keeps the nitrate of silver, with its disfiguring results, as a last resource, and then he administers it for nine months. A chapter on Rothbury, in Northumberland, as a health resort, gives a glowing account of the place as a bracing spot, especially adapted for city men who are "run down," and for patients in the early stages of phthisis. It seems a pity that the preface should be embellished with a "prodigious yarn" from a correspondent, as to the efficacy of some treatment, which reads like Mark Twain's prescription of two fair-sized whales as the amount of fish his patient should swallow.

**Taschenbuch der Massage.** Von Dr. Erich Ekgren. Pp. 90. Berlin: S. Karger. 1903.—This work, the author states, is written for medical men who have no time to study larger handbooks on the subject. It gives a short and accurate description of the different manipulations used in various cases, and also of the exercises most suitable as an auxiliary treatment. The chapter on gynæcological massage is treated somewhat more in detail.

**The Blood: How to Examine and Diagnose its Diseases.** By Alfred C. Coles, M.D. Second Edition. Pp. x., 286.

London: J. & A. Churchill. 1902.—This book not only deals with the subject in an extremely masterly way, but at the same time presents it in an easily understood and readable form. The subject is one which requires expert handling, and the details are so clearly explained that a beginner is often able, by following them, to get very good results. We have tried for some years many of the methods, and find them very satisfactory. There is one method of fixing films which we have found very useful, and which is not mentioned; viz., that of heating for fifteen minutes on a copper plate at the spot where a drop of xylol boils; this is especially useful with the triacid stain. The chief changes in this edition are in the sections on malaria and filaria, both of which are brought up to date and very fully dealt with. We can thoroughly recommend this as the best clinical book for general practice.

**Manual of Bacteriology.** By Robert Muir, M.D., and James Ritchie, M.D. Third Edition. Pp. xviii., 548. Edinburgh and London: Young J. Pentland. 1902.—The third edition of this excellent text-book contains many modifications and additions, which greatly enhance its value and bring it thoroughly up to date. The new matter includes chapters on the bacteriology of the air, soil, and water, and the standardisation of media. The group of "acid-fast" bacilli resembling the tubercle bacillus are also dealt with, and the subject of the "short" forms of diphtheria bacilli, or pseudo-diphtheria bacilli, is also discussed, but in rather too brief a form. The chapter on immunity has been remodelled so as to include recent observations, and prominence is here given to the theories of Ehrlich. New illustrations are introduced, and the illustrations generally are of a very high standard of excellence.

**Hygiene and Public Health.** By Louis Parkes, M.D., D.P.H., and Henry Kenwood, M.B., D.P.H. Second Edition. Pp. x., 763. London: H. K. Lewis. 1902.—The issue of a second edition of this work within a year of the appearance of the first edition is a sufficient guarantee of the popularity of the work. A few alterations of the text have brought it thoroughly up to date. The book is eminently suitable for the purpose of preparation for public-health examinations.

**Second Progress Report of "The Campaign against Mosquitoes in Sierra Leone."** By M. Logan Taylor, M.B. **Report of the Sanitary Conditions of Cape Coast Town.** By M. Logan Taylor, M.B. The University Press of Liverpool. 1902.—These two excellent reports should be in the hands of all persons residing in malaria-infected districts. They afford a clear and succinct account of the measures to be taken in order to suppress the multiplication of mosquitoes and insanitary conditions generally, which lead to the high sickness rate prevalent in tropical countries.

